# Prevalence and Assessment of Factors Associated with COVID-19 Vaccine Hesitancy in an Ethnic Minority Oncology Patient Population

**DOI:** 10.3390/vaccines10101711

**Published:** 2022-10-14

**Authors:** Matthew Lee, Emily Miao, Bruce Rapkin, Balazs Halmos, Viswanathan Shankar, Sanjay Goel

**Affiliations:** 1 Department of Oncology, Montefiore Einstein Cancer Center (MECC), Bronx, NY 10461, USA; 2 Department of Epidemiology and Population Health, Albert Einstein College of Medicine, Bronx, NY 10461, USA; 3 Department of Oncology, Rutgers Cancer Institute of New Jersey, New Brunswick, NJ 08903, USA

**Keywords:** COVID vaccine, vaccine hesitancy, ethnic minorities

## Abstract

Background: Complicating the COVID-19 pandemic are the healthcare disparities experienced by ethnic minorities, especially those with comorbidities including cancer. The introduction of COVID-19 vaccines has been instrumental in blunting the morbidity and mortality from the pandemic; however, vaccine hesitancy, particularly among ethnic minorities, has been a major concern. Thus, we sought to evaluate the knowledge and perspectives of COVID-19 and vaccines among our ethnic minority cancer patient population. Methods: Following an IRB approved protocol, questionnaires were completed by patients in a predominantly ethnic minority population at a single institution between 1 February and 30 June 2021. Included were any adult cancer patients with either a solid or hematologic malignancy. Results: Among the 84 patients that were offered the questionnaires, 52 patients responded, with a median age of 63.5 years. Overall, 36% were non-Hispanic Blacks and 30% were Hispanics; 65% were receiving active treatment for their cancer. Seventy-nine percent believed COVID-19 to be dangerous or harmful to them, 61% were concerned about the side effects, yet 65% considered COVID-19 vaccines as safe. Among the seven patients that refused the vaccine, (71%, *n* = 5) cited side effects and/or (57%, *n* = 4) believed that the vaccine was not needed. Overall, there was a significantly higher chance of being vaccinated if patients were receiving active cancer treatment, believed COVID-19 was harmful, or that the vaccine was safe, and knew COVID-19 was a virus. Conclusions: This exploratory study demonstrates that most ethnic minority cancer patients are receptive to vaccines, with a majority being vaccinated. However, we also discovered various reasons why this group of patients may not want be vaccinated, including concerns about side effects and perception that COVID-19 is not harmful. These findings can help us further understand the complex nature of vaccine hesitancy in ethnic minority cancer patients, and aid in developing future vaccine awareness strategies as the COVID-19 pandemic continues to evolve.

## 1. Introduction

The COVID-19 (Coronavirus Disease-2019) pandemic continues to pose a health risk globally and nationally within the United States (US), which has the highest caseload of COVID-19 in the world [1]. According to the Centers for Disease Control and Prevention (CDC), there is a disproportionate age-adjusted trend of rates of cases and deaths among racial and ethnic minorities [2,3,4]. Many studies have reported similar trends, with Black African and Latinx/Hispanic Americans having higher mortality rates compared with non-Hispanic White Americans and accounting for up to over 50% of COVID-19 related deaths [5,6,7,8,9,10,11,12,13]. This is then further exacerbated among patients with comorbidities such as diabetes, hypertension, heart failure, and importantly cancer [5,6,7,8,9,10,11,14,15].

Of all comorbidities, patients with malignancies have shown increased vulnerability with mortality up to 13–29% of COVID-19 related deaths [16,17,18,19,20]. Furthermore, clinical trials for COVID-19 have universally excluded patients with a history of cancer or those currently receiving treatment for cancer. Thus, it is imperative to understand the different reasons behind adherence to COVID-19 vaccination in ethnic minority cancer patients. However, COVID-19 vaccine hesitancy among racial and ethnic minority communities is influenced by complex factors that not only include safety concerns, socioeconomic factors, and access barriers but also misinformation, politicization, historical inequity, and medical mistrust [21,22,23,24], although these studies did not specifically evaluate cancer patients.

Previous studies surveying cancer patients were conducted mainly during December 2020–February 2021 when the vaccines were not widely available, in predominantly non-Hispanic White patient populations or in countries other than the United States (US) [25,26,27,28,29,30,31,32]. In these studies, overall, the main factors of increased hesitancy included similar reasons such as safety, side effects, process of development, lower education level, history of prior COVID-19, conservative political leaning, mistrust in health care systems, belief COVID is not harmful, and younger age [25,26,27,28,29,30,31].

Currently, only a few available studies have assessed vaccine hesitancy in cancer patients residing in the US, and in particular in Hispanic or Black cancer patients. In a cross-sectional study using online surveys of US cancer patients in December 2020, respondents who were unlikely/unsure of accepting the COVID-19 vaccine were more often Black compared to Non-Black and 55% less likely to accept a COVID-19 vaccine [33]. Major reasons behind hesitancy were questions of safety, inadequate information, mistrust of the government, seeing no reason for the vaccine, and fear of side effects [33,34,35]. The strongest predictors of vaccine uptake were plans to encourage vaccination of family, friends, community, and physician recommendation. On the other hand, the main reason for hesitancy was a fear of side effects [35]. A cross-sectional survey of adolescent and young adult cancer survivors of COVID-19 vaccine hesitancy, conducted from October 2020 to January 2021, showed higher odds of hesitancy if survivors were female or had a high school education or less, compared with survivors who were male or were college graduates or higher [36]. The only other US study involving mostly ethnic minority cancer patients was a study conducted in Puerto Rico [37]. Researchers showed that cancer patients were twice as likely than healthy individuals to be vaccinated, due to higher perceived COVID-19 susceptibility and severity [37].

Given the lack of studies assessing COVID-19 vaccine hesitancy in the US targeting specifically ethnic minority cancer patients, our study is the first to our knowledge to address this gap since vaccines were first widely distributed in New York City in the second phase including those with comorbidities starting in February 2021. The study aims to understand the various factors influencing vaccine hesitancy in this critical subgroup of high-risk patients and to help mitigate the existing health disparities. It was conducted in New York City, which was the initial epicenter of the COVID-19 pandemic in the United States and comprises one of the most diverse cities in the country. Among the five boroughs in New York City, the Bronx has the highest proportion of racial and ethnic minorities and the most persons living in poverty, along with one of the highest disproportionate burdens of COVID-19 cases, hospitalizations, and mortality rates as of January 2022 [14,38], highlighting further the need to evaluate vaccine perspectives in our population.

## 2. Methods

### 2.1. Study Design, Participants, and Setting

We conducted a cross-sectional survey of cancer patients in two hospitals within a health system with a predominantly ethnic minority population, between 8 March and 30 June 2021 during the COVID-19 vaccine roll out phase. The inclusion criteria were adults 18 years old or older, a diagnosis of any solid or hematologic malignancy at any disease stage, and currently receiving treatment (i.e., chemotherapy, immunotherapy, targeted therapy, or supportive care) and/or monitoring. An educational pamphlet about COVID-19 and COVID-19 vaccines was subsequently given to participants after completing the initial survey and a further survey was given on the same day with the same participant. All the patients who consented and participated in the survey were included. The Montefiore/Einstein Institutional Review Board approved the study.

### 2.2. Data Collection

All patients gave consent prior to any research activity. Included in the initial questionnaire were questions related to three broad categories: (1) demographics, which included self-reported race or ethnicity, gender, educational attainment, and employment status; (2) perceptions about COVID-19 and COVID-19 vaccines; and (3) basic knowledge about COVID-19 and COVID-19 vaccines. The survey questionnaire, educational pamphlet and post-survey are presented as supplemental material. Subsequent to the survey, the vaccination status was confirmed using electronic medical records cross-referenced with the NYS (New York State) immunization registry up to 1 January 2022.

*Outcome:* We considered the outcomes of self-reported vaccine status (during the survey) and electronic health record-confirmed vaccination status (6 months post-survey up to 1 January 2022).

*Knowledge:* Two composite scores were derived for assessing COVID-19-related knowledge and COVID-vaccine-related knowledge, to assess the question individually. Both knowledge scores ranged from 0–3.

### 2.3. Statistical Methods

Descriptive statistics were used to summarize numerically the demographic data, and the perception and knowledge of COVID-19 and COVID-19 vaccines. The normally distributed continuous scale variables were summarized using mean and standard distribution, and non-normally distributed variables were presented as median and range. The categorical variables were summarized as frequency counts and percentages. The association between vaccine status and patient characteristics, COVID-19-related knowledge, vaccine knowledge, and uptake motives was assessed using a t-test or chi-square test. Differences in beliefs about vaccine safety pre-and post-educational pamphlets were examined using McNemar or exact McNemar testing. A multivariable logistic regression model assessed the COVID-19 knowledge and vaccine knowledge score and its role on vaccination uptake. In addition, to understand the effects of patient characteristics on vaccination uptake (vaccinated during the survey and those who were vaccine naïve at the time of survey but were vaccinated subsequently), comparison with those who never took the vaccine was modeled using multinomial logistic regression. For all models, epidemiological confounders age, gender, and race were included along with those covariates that were significant at 30% alpha at the univariable model. All models were fitted using SAS 9.4 software.

## 3. Results

### 3.1. Patient Demographics

A total of 84 patients were offered questionnaires and 52 patients participated in the study. Of the participants, 58% (*n* = 30) were females. The participants’ average (standard deviation, sd) age was 63.5 (13.6) years; 36% reported themselves as Non-Hispanic Black (NHB), 30% as Hispanic, and 23% Non-Hispanic White (NHW). Almost 60% (*n* = 31) had a high school or lower level of education, and 34.6% reported being employed. Nearly 77% (*n* = 40) had solid malignancies, with over 65% of all participants reporting that they were currently receiving treatment for their cancer. Sixty-five percent of participants said their health was excellent or fair at the time of the survey. Twelve patients (23%) indicated they had had previous COVID-19 infections, of whom four (33%) required inpatient hospital admission. The prevalence of COVID-19 vaccination uptake during the survey was 40.4% (95%CI: 27, 54.9). The prevalence of vaccination uptake 6 months post-survey was 69.2% (95% CI: 54.9, 81.3). The distributions of patient characteristics by reported vaccination status are presented in Table 1.

### 3.2. Knowledge, Perceptions, and Attitude

**Figure 1:**
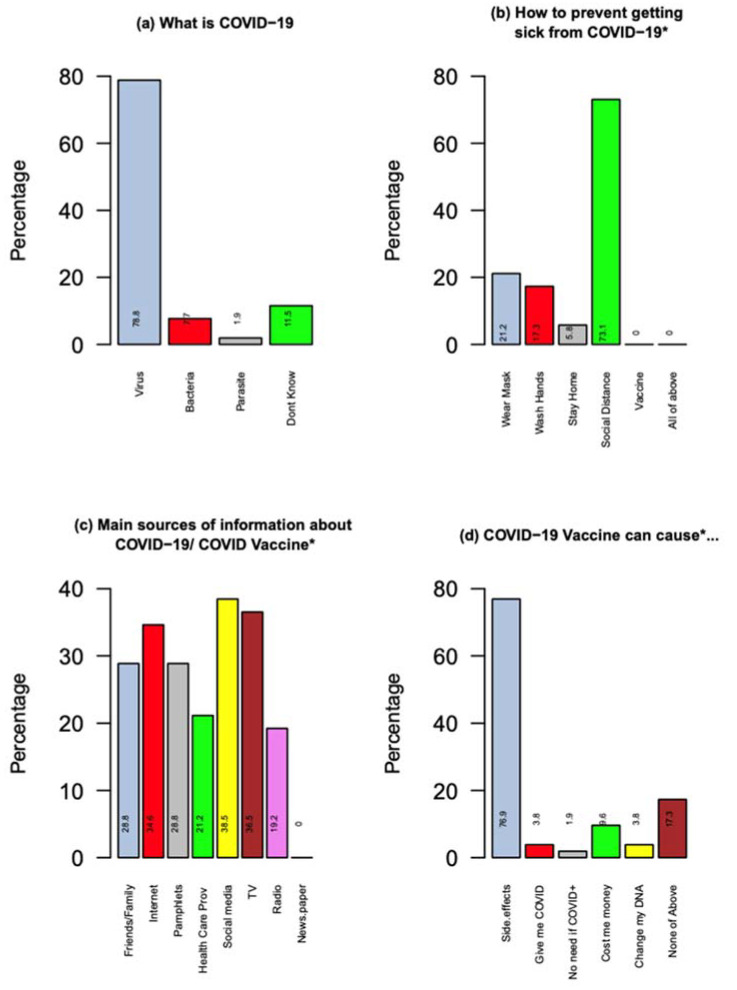
Distribution of Knowledge and Perceptions of COVID-19 and sources of information: (**a**,**b**) COVID knowledge, (**c**) sources, and (**d**) perception. * indicates multiple choice answers; the total percentages will be over 100%.

The overall estimated mean (sd) COVID-19-related knowledge score was 2.31 (0.81), while the vaccine-related knowledge score was 1.40 (0.99). The distribution of knowledge and perceptions is presented in Figure 1a–d. Nearly 56% of the participants reported a single source as their primary source of information about COVID-19 and COVID-19 vaccine; while the remaining 44% reported multiple sources ranging from two to seven. The participants indicated the following as their primary sources of information: television (38.5%), radio (36.5%), online internet sources (34.6%), family or friends (28.8%), social media (Twitter, Facebook, etc., 21.2%), and newspapers (19.2%). Regarding the information they received about the vaccine, irrespective of the correctness of the message, only 59.6% strongly agreed or agreed that they trusted the information they received about the vaccine.

As for the distribution of answers to COVID-19 knowledge related questions, almost 79% correctly identified that COVID-19 is a virus, while 10% believed it was a bacteria, fungus, or parasite. Another 73% of participants answered that face masks, hand washing, and COVID-19 vaccination were all ways of preventing COVID-19 infection and further spread.

In terms of the perceived threat of COVID-19, overall, 79% of participants believed COVID-19 to be dangerous or potentially harmful to them and 21% of participants believed it was not harmful or were unsure. Of those that believed COVID-19 to be not harmful, 63.6% were unvaccinated at the time of the survey. Post-survey, 54.6% of these participants remained unvaccinated in comparison to those that believed COVID-19 to be dangerous, with only 24.3% remaining unvaccinated (*p* = 0.0543).

Concerning belief in the efficacy of the COVID-19 vaccines, 46% believed vaccination would prevent them from getting COVID-19, while nearly 54% thought the vaccine would not prevent an infection (25%) or were unsure (28.8). Despite their beliefs, 46.3% (13/28) and 64.3% (18/28) had taken vaccines at the time of the survey and 6 months post-survey, respectively. Regarding perceptions of the vaccine’s safety, 61.5% were concerned about the side effects, but 65.4% still agreed that the COVID-19 vaccine was safe. A minority 4% answered they believed COVID-19 vaccines could give them COVID-19 and would change their DNA.

Nearly 41% of the participants believed that some ethnic and religious groups in the community faced difficulty accessing vaccinations. More than 86% (*n* = 45) reported that they would take the vaccine given an opportunity. Of those who mentioned that they would consider taking a vaccine (*n* = 45), their various reasons included protecting family or friends (62.2%), self-protection (55.6%), and ending the pandemic (48.9%). Of participants who declined to take the vaccine (*n* = 7), 71% (*n* = 5) mentioned side effects as their main reason.

### 3.3. Change in Knowledge and Uptake Attitude Post Informational Pamphlet

We examined vaccine uptake six months post-survey, through electronic health records; a total of 36 patients (69.2%) had taken the vaccine. An evaluation of the differences in knowledge of COVID-19 and COVID-19 vaccines resulted in statistically significant differences in those who were vaccinated post-survey compared with those unvaccinated, in their COVID-19 knowledge (*p* = 0.0005) and vaccine-related knowledge (*p* = 0.0033), although differences between these two groups were only marginally significant at the time of the survey, as summarized in Table 1. This association was then further examined in terms of participant characteristics, knowledge, and vaccine uptake at the time of the survey and 6 months post-survey, in univariable and multivariable logistic regression models, as summarized in Table 2.

The model suggests that vaccine uptake likelihood increased 2.1 times (*p* = 0.07) for a single unit increase in COVID-related knowledge adjusting for other covariates. Though statistically not significant, the likelihood of vaccine uptake at the survey was 2% higher per one unit increase in age. The chances of vaccine uptake were 1.8 and 1.9 times higher among Hispanics and African Americans than others. The patients reporting better health were 2.5 times more likely to take vaccines than those who did not (*p* = 0.16).

Furthermore, the adjusted model suggests that a single unit increase in vaccine-related knowledge increased the chance of vaccination over fivefold; similarly, a single unit increase in COVID vaccine knowledge increased three times the chance of vaccination, but this was not statistically significant.

We further examined the association of patient characteristics among those who had taken the vaccine at survey and post-survey, versus those who did not, by fitting a multinomial logistic regression. The results are presented in Table 3. The results suggest that adjusting for covariates, one unit increase in vaccine-related knowledge increased 6.5 times the probability of vaccination. Other significant variables were being male and having good health status. Being of a minority race was associated with a higher likelihood of getting vaccinated than other races but was not statistically significant.

Results after the distribution of the education pamphlet revealed that over 88% (*n* = 46) indicated that they learned more about COVID-19 after reading the pamphlet, while for patients (7.7%) said no, and two (3.9%) were unsure.

## 4. Discussion

The development and authorization of effective COVID-19 vaccines in less than one year prompted wide deployment in the United States, but was also met quickly with as hesitancy and refusal. This concern is further amplified if a patient with COVID-19 is not only unvaccinated but also has comorbidities such as cancer, predisposing them to higher risk of morbidity and mortality. COVID-19 vaccine hesitancy has been studied previously in cancer patients worldwide [25,26,27,28,29,30,32,39,40,41,42,43,44] and in the United States [31,33,35,36,45], including multiple studies of ethnic minorities in the US [21,22,23,24,46,47,48,49,50,51], but no known research exists specifically examining ethnic minority cancer patients and their views on the COVID-19 vaccine. This study helps address this gap in the knowledge by uniquely examining two different time-points of vaccination status 6-months post-initial survey, employing a vaccine knowledge score composite and exploring a brief educational intervention to raise patients’ awareness, to gain valuable insight into this distinctly disadvantaged population in the US.

In our study on cancer patients, 95% of the respondents were either Black or Hispanic, with 65% vaccinated and actively receiving treatment for their cancer during the crucial time of the initial rollout of COVID-19 vaccines in New York City. Overall, a large majority of our patients—79%—perceived that COVID-19 was risky and dangerous, and 65% considered the vaccines to be safe, similar to prior results in cancer patient studies [25,28,29,30,33,35,41,42,43,44]. Other self-reported reasons for being vaccinated stated by our participants included protecting themselves, family, and friends, the collective good of ending the pandemic, and trust in the vaccine and science; again, these were very similar to previous studies in cancer patients and ethnic minorities [23,25,28,29,31,35,50]. Participants who had self-reported a fair or excellent health at the time of the survey were seen to have a significantly higher likelihood of being vaccinated, 22 times more likely compared with those who self-reported poor health, according to our multivariable logistic regression and multinomial logistic regression models (Table 2 and Table 3); this represents another factor to consider when examining future vaccine uptake studies in this population. Lastly, our study showed that male participants were more likely than females to be vaccinated (Table 2 and Table 3), a finding similar to previous studies [23,29] which potentially warrants a closer look into as to why there may be differences in gender for vaccination uptake in this group.

In contrast, when examining those who were unvaccinated or refused the vaccine, their main reasons were concerns about side effects (71%), that it was not needed (57%), and mistrust in the science of the vaccine (14%), which are similar to results from other studies [25,28,29,30,33,35,40,42,43,44]. Among participants who answered that COVID-19 was not harmful (21% of the total, *n* = 11), 64% remained unvaccinated at the time of the initial survey. Interestingly, 6 months after the initial survey, 54.6% of these patients remained unvaccinated (*p* = 0.0543). This indication of low perceived threat from COVID-19 as a negative predictor of vaccination has been demonstrated previously [23,28,29,40,44] and may suggest a persistent belief even as the pandemic continues and the vaccines are more widely available, and represents a possible important factor for interventions to target.

Knowledge about COVID-19 was also found to be an important predictor and component of COVID-19 vaccine hesitancy in our study population. Over 70% of participants were able to answer correctly questions of COVID-19-related knowledge and about the vaccine itself, with significantly higher likelihood of vaccination based on composite scores from their answers (Table 2 and Table 3). Intriguingly, this is driven by higher COVID-19 vaccine knowledge but not necessarily on basic knowledge of COVID-19 itself. A prior study showed that poor knowledge about vaccines was a negative predictor of vaccination [7]; this may represent a future area of research to explore further this discrepancy and whether efforts for increasing vaccine knowledge could be more helpful.

Another area of interest was to examine where patients received their information from, and the impact of their educational backgrounds. Our study demonstrated that participants encountered a wide range of sources of information, from television and radio to online sources such as the internet and social media, along with family and friends, similar to prior research [43]. Additionally, a positive predictor of vaccination uptake as seen previously was having access to information about COVID-19 vaccines [26,33,44], and trusting medical advice on vaccination [29,35,39]. Yet, only 60% trusted the information they had received, reflecting wide prior research [25,27,28,29,33,43,45], and highlighting concerns around the need to acquire more information [21,46,47,49,50,51]. These results present another avenue where increased awareness of the issue can help alleviate this problem, and suggest that a focus on correcting misinformation may help increase vaccination rates. Vaccine awareness programs, public participation, and reminder–recall approaches based on WHO guidelines have been other proposed solutions to increase vaccine confidence [38].

In terms of education, vaccination uptake was only significantly higher in those with a high school education or higher in the 6-month follow-up when vaccination status was examined again, which was similar to COVID-19 knowledge scores (Table 1). However, education did not play a significant role in the odds of vaccination in our multivariable models, unlike its significance reported in other studies showing higher education as a positive predictor of vaccination [25,36] and lower education as a negative predictor [23,25,31,40,44]. This socioeconomic determinant may still contribute to vaccination, but further expansion of our study would need to demonstrate that.

The long-standing healthcare disparities have been stated in various studies on COVID-19 vaccine hesitancy in ethnic minority groups, referring to accessibility and equity concerns, mistrust in the healthcare system and government [21,46,47,49,50,51], structural, interpersonal, historical, and contemporary racism and anti-immigration [47,48,50], barriers in transport and technology [21,46,47,48], housing insecurity [24], and English as a second language [47]. In our study, about 41% of participants felt that some ethnic and religious groups in the community had difficulty getting vaccinations, and only 60% trusted the information they received, although neither employment nor race and ethnicity significantly determined vaccine uptake in our study. These all represent areas of socioeconomic determinants that can play roles in vaccine hesitancy, and are areas for improvements.

As evidenced by our study, future interventions to increase vaccine uptake should emphasize the safety and efficacy of vaccines, highlighting its protective effects for both individuals and their families, as well as the additional protection vaccines confer [52]. Furthermore, given the mistrust that is evident in ethnic minorities, there have been prior studies on effective interventions including those from community or faith-based stakeholders [53,54] to help raise vaccination uptake. Starting early conversations, addressing side-effect concerns, and maintaining transparency are all crucial to encouraging vaccine uptake. Other interventions such as vaccine awareness programs, public participation, and reminder–recall approaches based on WHO guidelines have been proposed as solutions to increase vaccine confidence [38].

Ongoing research with this population can further expand on the knowledge gained in this study, highlighting that knowledge of COVID-19 vaccine itself plays a role in uptake of the vaccine. Exploratory interventions such as a simple paper pamphlet can have the potential to help increase COVID-19 knowledge but need further validation and expansion, and more modern interventions with webinars can be effective [54]. There is a need for focus groups, patient-centered communication approaches [53,55], and an expansion of questions to include relations of political participation and beliefs [56] and explore other factors that may relate to vaccine hesitancy. In the current era of increasing booster vaccination in the cancer population, there may be related issues similar to the initial wave of vaccinations.

Our study is noteworthy for its uniqueness in that it only studied patients with cancers as their main life-threatening illness. However, is has certain limitations deserving discussion. We believe that the main limitation is that the study was based mainly on a paper questionnaire administered in person, which limited the number of surveys that could be distributed and resulted in a smaller sample size. A second limitation was the time of the survey in relation to the overall trajectory of the pandemic and the global response. The surveys were initially distributed when vaccines were first made available to the public. Since then, more cancer patients have been vaccinated compared with the cohort we analyzed. For example, according to reports from February 2022, 90% of adults in the Bronx, NY have received at least one dose and 80% are fully vaccinated; which although lower than the average New York City-wide rate, is still a relatively high vaccination rate.

However, a recent study in 2022 showed that although there are now higher vaccination rates among non-Hispanic Blacks and Hispanics, which have reduced mortality, there remains a high disparity in mortality between these ethnic groups and non-Hispanic Whites [57]. Despite these high vaccination rates, as many as 10% of adults are unvaccinated and if they have cancer can have a higher risk of mortality than a vaccinated cancer patient. Thus, improving vaccination rates for all cancer patients should continue to remain a goal.

## 5. Conclusions

Although a majority of cancer patients such as those in our study are vaccinated, which is consistent with prior reports [29,30,32,41,42,43], cancer patients have been an under-represented group in vaccine clinical trials, especially ethnic minorities. Therefore, it may be justified for these patients to have safety concerns and skepticisms. This study has explored various factors and concerns of vaccine hesitancy and refusal, and identified potential areas for improving adherence, in a predominantly ethnic minority cancer population, at two different time points and follow up. This population not only had the healthcare disparities and socioeconomic factors associated with ethnic minority patients, but the added affliction of cancer.

These findings illustrate that the main factors motivating patients towards vaccination were safety perceptions, with a higher likelihood of vaccination if participants felt it was safe but interestingly also a higher likelihood of being vaccinated if they answered they were concerned about the side effects of the COVID-19 vaccine. This could highlight that even though cancer patients may be concerned about the side effects, they still feel vaccination is safe overall and are willing to be vaccinated because they still feel COVID-19 to be harmful to them. This may indicate that motivation towards vaccination in this population still involves these factors, but that they do not ultimately prevent patients from receiving the vaccination. Additionally, self-reported status, trust in information they receive, self-perceived harm, self-protection, protection of their family, friends, and individual COVID-vaccine knowledge all were important key factors for vaccine uptake found in our study. These represent possible targets for future interventions as COVID-19 continues. With evolving variants and as new vaccines become available, strategies to increase vaccine uptake will continue to be important, especially in historically underrepresented marginalized patient populations such as ethnic minority cancer patients.

## Figures and Tables

**Table 1:** Distribution of patient characteristics by vaccination status (Initial and post-survey).

	Initial Survey			Post-Survey		
Characteristics	Non-Vaccine (*n* = 31)	Vaccine (*n* = 21)	*p*-Value	Non-Vaccine (*n* = 16)	Vaccine (*n* = 36)	*p*-Value
Age (years) Mean (sd)	63.0 (14.8)	64.4 (11.8)	0.7200 †	65.6 (15.2)	63.7 (12.9)	0.4921 †
**Gender**						
Female	18 (60.0)	12 (40.0)	0.9474	12 (40.0)	18 (60.0)	0.1311 *
Male	13 (59.1)	9 (40.9)				
**Race/Ethnicity**						
Hispanic	10 (62.5)	6 (37.5)	0.7323	5 (31.3)	11 (68.8)	0.8428
NH-African American	10 (52.6)	9 (47.4)		5 (26.3)	14 (73.7)	
Others	11 (64.7)	6 (35.3)		6 (35.3)	11 (64.7)	
**Education**						
≤HS	19 (61.3)	12 (38.7)	0.7649	14 (45.2)	17 (54.8)	0.0070 *
>HS	12 (57.1)	9 (42.8)		2 (9.5)	19 (90.5)	
**Employment**						
Employed	10 (55.6)	8 (44.4)	0.6672	4 (22.2)	14 (77.8)	0.5286
Unemployed/Homemaker	21 (61.8)	13 (38.2)		12 (35.3)	22 (64.7)	
**Health Status**						
Excellent/Fair	18 (52.9)	16 (47.1)	0.2394 *	8 (23.5)	26 (76.5)	0.1200
Poor	13 (72.2)	5 (27.8)		8 (44.4)	10 (55.6)	
**Current Cancer Trt**						
Yes	23 (59.0)	16 (41.0)	0.8704	9 (23.1)	30 (76.9)	0.0666
No	8 (61.5)	5 (38.5)		7 (53.8)	6 (47.2)	
**COVID Infection**						
Yes	9 (75.0)	3 (25.0)	0.3184 *	2 (16.7)	10 (83.3)	0.3010
No	22 (55.0)	18 (45.0)		14 (35.0)	26 (65.0)	
**COVID-19 Knowledge** Mean (sd)	2.16 (0.90)	2.52 (0.60)	0.0874 †	1.75 (0.86)	2.56 (0.65)	0.0005 †
**COVID Vaccine knowledge** Mean (sd)	1.29 (0.90)	1.57 (0.87)	0.3224 †	0.81 (0.98)	1.67 (0.89)	0.0033 †

* Fisher’s Exact Test, † Student *t*-test.

**Table 2:** Univariable and multivariable logistic regression models at survey time and 6 months post-survey on vaccine status.

Logistic Regression Model for Vaccine Status at Survey (21 vs. 31)				
	Univariable Model		Multivariable Model	
Variables	OR (95% CI)	*p* Value	aOR (95% CI)	*p* Value
**Age (years)**	1.01 (0.97, 1.05)	0.7140	1.01 (0.97, 1.06)	0.5468
**Gender**				
Male	1.04 (0.34, 3.19)	0.9473	0.92 (0.26, 3.37)	0.9089
Female	1.00		1.00	
**Race/Ethnicity**				
Hispanic	1.10 (0.27, 4.55)	0.8953	1.85 (0.38, 8.95)	0.4458
African American	1.65 (0.43, 6.31)	0.4645	2.16 (0.49, 9.48)	0.3080
Other	1.00		1.00	
**Education**				
≤HS	1.00	0.9883		
>HS	1.18 (0.33, 4.21)			
**Employment**				
Employed	1.27 (0.34, 4.77)	0.8866		
Unemployed	1.00			
**Health Status**				
Good	2.28 (0.59, 10.04)	0.2931	2.93 (0.73, 11.75)	0.1289
Fair/poor	1.00		1.00	
**COVID-19 Knowledge Score**	1.81 (0.83, 4.33)	0.1539	1.80 (0.69, 4.66)	0.2292
**COVID-19** **Vaccine Knowledge Score**	1.33 (0.73, 2.49)	0.3932	1.33 (0.64, 2.80)	0.4471
Logistic regression model for vaccine status 6-months post-survey (36 vs. 16)				
**Age (years)**	0.98 (0.94, 1.03)	0.4851	0.97 (0.91, 1.03)	0.3489
**Gender**				
Male	3.00 (0.81, 11.08)	0.0994	11.59 (1.04, 129.16)	0.0463
Female	1.0		1.00	
**Race/Ethnicity**				
Hispanic	1.20 (0.28, 5.12)	0.8055	4.99 (0.37, 67.16)	0.2253
African American	1.53 (0.37, 6.35)	0.5604	3.36 (0.39, 29.20)	0.2719
Other	1.0		1.0	
**Education**				
≤HS	1.00	0.0128		
>HS	7.82 (1.55, 39.52)			
**Employment**				
Employed	1.91 (0.51, 7.11)	0.3352		
Unemployed	1.00			
**Health Status**				
Good	2.60 (0.77, 8.82)	0.1253	22.41 (1.95, 257.07)	0.0125
Fair/poor	1.00			
**COVID-19 Knowledge Score**	3.79 (1.63, 8.82)	0.002	3.10 (0.79, 12.25)	0.1062
**COVID-19** **Vaccine Knowledge Score**	2.81 (1.32, 5.96)	0.0072	5.02 (1.33, 18.92)	0.0171

aOR—adjusted odds ratio.

**Table 3:** Multinomial logistic regression model on vaccination status pre-survey vaccination and 6 months post-survey vaccination.

	Post-Survey Vaccination vs. None (15 vs. 16)		Pre-Survey Vaccination vs. None (21 vs. 16)	
Variables	aOR (95% CI)	*p* Value	aOR (95% CI)	*p* Value
Age (years)	0.94 (0.87, 1.02)	0.1285	0.99 (0.92, 1.06)	0.7080
**Gender**				
Male	21.5 (1.32, 348.33)		7.16 (0.56, 90.95)	
Female	1.0	0.0308	1.0	0.1291
**Race/Ethnicity**				
Hispanic	3.65 (0.18, 73.19)	0.3978	3.57 (0.23, 54.61)	0.3607
African American	3.02 (0.22, 41.67)	0.4095	3.26 (0.33, 32.16)	0.3110
Other				
**Education**				
≤HS	1.00		1.00	
>HS	5.55 (0.52, 59.20)	0.1557	1.78 (0.20, 15.84)	0.6055
**Health Status**				
Good				
Fair/poor	20.20 (1.21, 338.16)	0.0365	20.92 (1.60, 274.09)	0.0205
**COVID-19 Knowledge Score**	1.83 (0.32, 10.63)	0.4974	2.64 (0.57, 12.09)	0.2125
**COVID-19** **Vaccine knowledge Score**	6.51 (1.33, 31.84)	0.0207	4.61 (1.16, 18.39)	0.0305

aOR—adjusted odds ratio.

## Data Availability

The data presented in this study are available on request from the corresponding author.
